# When Attitudes Become Obstacles: An Exploratory Study of Future Physicians’ Concerns about Reporting Child Maltreatment

**DOI:** 10.3390/children10060979

**Published:** 2023-05-31

**Authors:** Morgan E. Dynes, Stephne S. Rasiah, Michele Knox

**Affiliations:** 1 Department of Psychiatry, University of Toledo, Toledo, OH 43614, USA; michele.knox@utoledo.edu; 2 College of Medicine and Life Sciences, University of Toledo, Toledo, OH 43614, USA; stephne.rasiah@rockets.utoledo.edu

**Keywords:** child maltreatment, mandated reporting, physicians, medical students, residents

## Abstract

Background: Child maltreatment (CM) makes up a significant portion of events under the larger umbrella term of adverse childhood experiences (ACEs). Therefore, we need to develop a competent healthcare workforce that is prepared to assess and report CM in order to create a comprehensive framework for assessing and addressing ACEs. The objective of the present study was to examine the obstacles to reporting CM among a sample of future physicians. Methods: Two samples of medical students and residents (*N* = 196) completed the Healthcare Provider Attitudes Toward Child Maltreatment Reporting Scale and rated how likely they would be to report suspected CM. Results: Medical students were found to have more negative feelings about and perceive more obstacles to reporting CM compared to residents in our sample. Scores on the Reporting Responsibilities subscale were not significantly associated with increased likelihood of reporting CM. However, lower scores on the Concerns about Reporting subscale were related to an increased likelihood of reporting CM. Conclusions: Future physicians who perceived fewer obstacles to reporting CM reported being more likely to report suspected CM. Misinformed fears about outcomes such as retaliation, removal of the child from the home, and being sued may interfere with future physicians’ adherence to mandated reporting responsibilities. Efforts should be made early in physician education to identify and address common myths and misconceptions around mandated reporting and its outcomes.

## 1. Introduction

Healthcare treatment providers, including physicians, often provide care for children who endure maltreatment and household dysfunction. Adverse childhood experiences (ACEs) are stressful experiences that occur during childhood and directly affect the child and/or the family environment [1]. They include physical, sexual, or emotional abuse, neglect, and difficulties experienced by caregivers that can influence the environment in which the child grows up (e.g., intimate partner violence, divorce, parent incarceration, substance abuse, or mental health problems) [2]. The potential impacts of ACEs are physiological, behavioral, and psychological, and they yield poorer outcomes across multiple health domains [2,3]. Researchers, in follow-up studies, have demonstrated a graded relationship between the number of ACEs and later-life risk for suicide, alcoholism, illicit drug use, depression, diabetes, heart disease, stroke, cancer, and premature mortality [4,5].

The American Academy of Pediatrics recommended in 2012 that pediatricians screen for ACEs and develop approaches to support children exposed to potentially toxic stressors [6,7]. However, there are controversies surrounding the routine screening of ACEs, including debates such as which ACEs to screen for and when, thresholds for referring to services after screening, and the impact of assessing these sensitive experiences on the patient and their family [8,9,10]. When screening procedures for ACEs are developed, the mandated reporting of child maltreatment (CM) by healthcare professionals is a necessary consideration. Therefore, behaviors and attitudes related to mandated reporting of CM should be seen as a critical component of developing thoughtful screening practices for ACEs and providing trauma-informed healthcare to children and families.

While there is increased interest in screening for ACEs, 61% of pediatricians reported that they did not screen for ACEs, and only 2% reported that they were familiar with the ACEs study in a national survey [10,11,12]. There is a multitude of reasons for low rates of screening for ACEs, including the already-extensive recommendations for content areas needing to be covered in healthcare visits, lack of time and confidence in discussing trauma-related issues, and reluctance to receive feedback necessitating referrals to child protective services. Challenges to physician reporting of child maltreatment are well documented, and researchers have found that child maltreatment cases often go unreported [13]. Twenty to forty percent of mandated reporters across healthcare professions have failed to report at least one incident in their career despite having concerns that abuse may have occurred [14,15]. While the issue of under-reporting of CM is multifactorial, the inadequate training of mandated reporters is one important contributor. Researchers have documented that many medical professionals feel undertrained and unprepared to identify and report child maltreatment [14,16].

Furthermore, issues with reporting CM have been investigated with other child-serving professions. A study assessing factors influencing the reporting of child maltreatment in a sample of nurses identified beliefs and attitudes such as low faith in the child protection system, beliefs that there are various organizational and individual barriers to reporting, and distrust that reporting will benefit the child and family [17]. In a study of student and professional mental health practitioners [18], an educational intervention designed to improve reporting of child maltreatment included a group discussion format and techniques to promote learning about how personal biases may influence reporting behavior. The curriculum was found to be effective with mental health professionals and trainees. Another study addressing child maltreatment also studied trainees—in this case, pediatrics residents [19]. The authors reported that residents, as opposed to those later in their careers, are more open to new ideas and approaches. Thus, training that takes place earlier in an academic/career path may be most effective. This may be especially true with training that addresses attitudes and beliefs that, once ingrained, would be difficult to alter. Healthcare providers are critical to the identification and amelioration of adverse childhood experiences among youths and their families. While there are complexities to consider, the reporting of CM could be seen as an intervention strategy that can begin to mitigate the negative effects of abuse and neglect. Therefore, we need to develop a competent workforce that is prepared to assess and report CM in order to develop well-thought-out screening and referral practices to address ACEs. To accomplish this goal, we need to increase our understanding of the barriers to reporting and assess future physicians’ comfort with reporting CM. Enhancing our understanding of these obstacles could inform educational and training strategies in order to improve practitioners’ reporting practices [17]. The objective of the present study was to examine the obstacles to reporting child maltreatment among a sample of future physicians. The Healthcare Provider Attitudes Toward Child Maltreatment Reporting Scale (HPATCMRS) was developed to identify such obstacles and has been used to further understand the problem of under-reported CM [20]. The HPATCMRS is a 21-item measure that identifies attitudes and concerns that could pose obstacles to the mandated reporting of CM [20,21]. Researchers have utilized this scale to evaluate the outcomes of education and training designed to improve adherence to mandated reporting in a variety of healthcare professional samples [22,23]. It was hypothesized that medical students would endorse more negative feelings about and perceive more obstacles to reporting CM compared to residents. It was further hypothesized that higher knowledge about and commitment to reporting child maltreatment (as indicated by scores on the Healthcare Provider Attitudes Toward Child Maltreatment Reporting Scale’s Reporting Responsibilities subscale) would be associated with an increased likelihood of reporting suspected CM. It was hypothesized that fewer negative feelings about and lower perceived obstacles to reporting child maltreatment (as indicated by scores on the Healthcare Provider Attitudes Toward Child Maltreatment Reporting Scale’s Concerns about Reporting subscale) would be associated with an increased likelihood of reporting suspected CM.

## 2. Materials and Methods

### 2.1. Participants

Eighty-four participants consented to participate in the study and attended a 1-h didactic lecture on CM advocacy, reporting, and treatment. Of these, 51 were family medicine residents, 20 were pediatrics residents, 6 were neurology residents, 1 was a psychiatry resident, and 6 were medical students. When asked the gender with which they self-identified, 23 reported “male”, 48 reported “female”, 4 indicated that they preferred not to say, and 9 did not respond. The ethnic classifications reported by the participants included 1 American Indian/Alaska Native, 18 Asian, 5 Hispanic/Latinx, 47 Caucasian, 1 Hawaiian Native, and 12 Other. The age of this sample ranged from 23 to 61 years, with an average of 29.64 years (*SD* = 6.03).

In addition, for the purposes of comparing medical students’ and residents’ scores on the HPATCMRS, a sample of 120 first-year medical students was added to the sample. Demographic information was not collected for this subsample. This subsample’s data were reported in a previous study on measure development/factor analysis of the HPATCMRS scale [20].

Following analysis, 196 of the 204 total participants yielded valid survey results for analysis. Eight participants were not included in final analyses due to these participants not completing all items on the survey.

### 2.2. Measures

Every participant was asked to respond to the item “Right now, how likely would you be to report child maltreatment if you suspected, but did not know for sure, that a child has been maltreated?” Their responses ranged on a 5-point Likert scale from 1 = very unlikely to 5 = very likely. All participants also completed the Healthcare Provider Attitudes Toward Child Maltreatment Reporting Scale (HPATCMRS). The HPATCMRS was originally developed to identify attitudes and concerns that could pose obstacles to the mandated reporting of child maltreatment [20]. Additionally, this scale is used to evaluate the outcomes of education and training designed to improve adherence to mandated reporting. The scale comprises a total of 19 items that make up 2 subscales. Most items of the HPATCMRS are rated on a 5-point Likert scale: strongly agree = 5, agree = 4, neutral = 3, disagree = 2, and strongly disagree = 1. However, some are reverse-scored. See Table 1 for items that are reverse-scored, as indicated by asterisks.

The 10-item HPATCMRS Reporting Responsibilities subscale measures knowledge about and commitment to reporting child maltreatment, with higher scores indicating more knowledge about and commitment to reporting CM. The scores of this subscale can range from 10 to 50. The 9-item HPATCMRS Concerns about Reporting subscale measures feelings about and perceived obstacles to reporting child maltreatment, with higher scores indicating less apprehension about and fewer obstacles to reporting. The scores on this subscale can range from 9 to 45. The items included in each subscale are listed in Table 1. The subscales were identified via factor analyses, and internal consistency reliability analyses of the subscales identified an alpha coefficient of 0.85 for the 10-item Reporting Responsibilities subscale and 0.82 for the 9-item Concerns about Reporting subscale [20].

### 2.3. Procedures

The institutional review board of this study’s authors’ institution reviewed and approved the study protocol. Informed consent was obtained from the first cohort (84 participants). However, the retrospective study with data collected from a sample of 120 first-year medical students was designated exempt by the institutional review board; therefore, signed informed consent was not obtained. For the first cohort of 84 participants, the surveys were delivered using Qualtrics—an online survey platform. Participants were eligible to participate in the study if they were current residents or medical students. This cohort included participants from nine resident training programs across the United States. These participants were given the HPATCMRS questionnaire prior to completing a 1-h didactic lecture on CM advocacy, reporting, and treatment. Data were collected over a 10-month period from February 2022 to December 2022. Additional archival paper surveys were analyzed from a 2017 cohort of 120 first-year medical students. These participants were given the HPATCMRS questionnaire in the form of a paper survey prior to completing an elective course on child maltreatment advocacy, reporting, and treatment.

## 3. Results

The internal consistencies for the subscales in the present study were as follows: Reporting Responsibility Subscale Cronbach’s alpha = 0.72, Concerns about Reporting Subscale Cronbach’s alpha = 0.83. The entire combined (*N* = 196 valid scores) sample’s mean score on the Reporting Responsibility Subscale was 37.69 (*SD* = 3.77). The mean score on the Concerns about Reporting Subscale was 28.06 (*SD* = 6.03). Independent-samples *t*-tests were conducted to compare medical students’ mean scores with residents’ mean scores on both HPATCMRS subscales. The results indicated no significant difference on the Reporting Responsibilities subscale. In contrast, there was a significant difference on the Concerns about Reporting subscale that reflected medical students’ significantly lower mean score (x = 26.21 (SD = 4.95) vs. x = 30.82 (SD = 6.46); t(190) = 5.59, *p* < 0.001) on this subscale. This finding represents a large effect size (Cohen’s d = 0.82) and suggests that, compared to residents, medical students have more negative feelings about and perceive more obstacles to reporting child maltreatment. See Table 1 for medical students’ and residents’ mean item scores on all HPATCMRS items.

Mean item scores below 3 indicate decreased knowledge and reduced commitment on the Reporting Responsibilities subscale, and increased apprehension and perceived obstacles on the Concerns about Reporting subscale. The Reporting Responsibilities subscale items with such low scores for the resident portion of the sample included (3 items total) “I believe the current system for reporting CM is effective in addressing the problem”, “I lack confidence in the authorities to respond effectively to reports of CM”, and “I will consult with an administrator/supervisor before I report CM”. The Concerns about Reporting subscale items with mean scores below 3 for the resident portion of the sample included (2 items total) “A CM report can cause a parent to become more abusive toward the child” and “There is a lot of sensitivity associated with reporting CM”.

The Reporting Responsibilities subscale items with mean scores below 3 for the medical student portion of the sample included (3 items total) “I believe the current system for reporting child maltreatment is effective in addressing the problem”, “I lack confidence in the authorities to respond effectively to reports of child maltreatment”, and “I will consult with an administrator/supervisor before I report child maltreatment”. The Concerns about Reporting subscale items with mean scores below 3 for the medical student portion of the sample included (5 items total) “I would be apprehensive to report child maltreatment for fear of family/community retaliation”, “I feel emotionally overwhelmed by the thought of reporting child maltreatment”, “ I would not report child maltreatment if I knew the child would be removed from the home/family”, “I would consider not reporting child maltreatment because of the possibility of being sued”, and “It is a waste of time to report child maltreatment because no one will follow up on the report”.

In order to examine relationships between concerns about reporting and the likelihood of reporting, and between reporting responsibilities and the likelihood of reporting, one-way ANOVAs were conducted, with the participants’ responses (1 = very unlikely to 5 = very likely) to the question “Right now, how likely would you be to report child maltreatment if you suspected, but did not know for sure, that a child has been maltreated?” as the factor categories, and the subscale scores as dependent variables. The results are listed in Table 2.

Group differences were not identified on the Reporting Responsibilities subscale scores. However, the groups differed significantly on scores on the Concerns about Reporting subscale (F(3, 79) = 11.56, *p* < 0.001; eta-squared = 0.24)), representing a small effect size. The direction of the group differences suggests that those with fewer concerns about/perceived obstacles to reporting would be more likely to report suspected maltreatment.

## 4. Discussion

The objective of the present study was to examine the obstacles to reporting child maltreatment among a sample of future physicians. It was hypothesized that medical students would endorse more attitudes that interfere with the adherence to mandated reporting of CM compared to residents. Our results partially supported this hypothesis. Specifically, medical students were not found to have less knowledge or commitment to reporting; however, they did report more negative feelings and perceived more obstacles to reporting CM compared to residents in our sample.

It was also hypothesized that higher knowledge about and commitment to reporting CM (as indicated by scores on the HPATCMRS Reporting Responsibilities subscale) would be associated with an increased likelihood of reporting suspected CM. This hypothesis was not supported. Lastly, fewer negative feelings about and perceived obstacles to reporting CM (as indicated by scores on the HPATCMRS Concerns about Reporting subscale) were found to be significantly associated with an increased likelihood of reporting suspected CM. This finding supported our third hypothesis. Below, we discuss the implications of our findings and directions for future research.

One potential reason that the Reporting Responsibilities subscale scores were not significantly associated with the likelihood of reporting suspected CM could be that future physicians know “the right answers” to items on this subscale (e.g., “I plan to report CM when I suspect it”, or “CM reporting guidelines are necessary for physicians”) or are aware of their responsibilities for reporting CM in theory, but that knowledge may not influence their real-world likelihood of reporting CM when they suspect it. This may be particularly relevant in the face of misinformed fears that operate as obstacles towards reporting. Specifically, the results of the present study indicate that these include beliefs that a report can cause a parent to become more abusive toward the child, that the child may be removed from the home, and that there may be retaliation or lawsuits following the report. These findings indicate that the training of future physicians should include education about the actual likelihood of such events, as well as state laws protecting mandated reporters.

The lack of statistical significance in the relationship between the Reporting Responsibilities subscale and the likelihood of reporting CM could suggest how to focus the limited time allotted for mandated reporting in future physicians’ education. This study’s findings indicate that focusing on identifying and addressing feared outcomes around reporting could be most impactful for helping to increase the likelihood of future physicians reporting CM.

Although concerns about reporting responsibilities did not relate to the likelihood of reporting, it is still important to note that both medical students and residents reported beliefs that the CM reporting system is not effective, and that they lack confidence in the authorities regarding responding to CM reports. These beliefs are suggestive of poor faith (justified or not) in child protective services among future physicians.

This study has several limitations. It should be noted that the residents in our sample completed surveys online via Qualtrics, while the majority of the medical students subsample completed paper surveys. This difference in survey administration may have impacted their responses. Future studies should use a consistent method of gathering data. Furthermore, although the resident subsample was collected from multiple sites across the United States, the medical student subsample was primarily obtained from a single educational institution. Therefore, the medical student results may not be as generalizable to the larger population of medical students across the country. Furthermore, our study did not obtain demographic information for the subsample of medical students. Lacking this demographic information prevented this study from fully describing this subsample or examining demographic differences among the subsample groups. Lastly, the outcome variable of likelihood of reporting suspected CM was measured using only one item. Future studies should include a comprehensive measure of likelihood or actual reporting of CM.

The results from this study shed some light on how fears around the outcomes of reporting suspected CM could influence the emerging healthcare workforce’s adherence to mandated reporting of CM. Training methods used with future physicians should focus on how medical students’ education can be tailored to explore and address these fears directly, and on teaching effective methods for gathering information and assessing for CM.

## Figures and Tables

**Table 1:** Means and standard deviations for residents’ and medical students’ responses to the Healthcare Provider Attitudes Toward Child Maltreatment Reporting Scale items.

Healthcare Provider Attitudes toward Child Maltreatment Reporting Scale Item	Residents			Medical Students		
	*N*	*M*	*SD*	*N*	*M*	*SD*
Reporting Responsibilities						
I would still report CM even if my supervisors disagreed with me	77	3.32	(1.032)	120	3.28	(0.881)
I lack confidence in the authorities to respond effectively to reports of CM *	77	2.97	(1.112)	120	2.81	(0.901)
I plan to report CM when I suspect it	78	4.49	(0.849)	120	4.28	(0.621)
Reporting CM can enable services to be made available to children and families	78	4.13	(0.812)	120	4.08	(0.637)
I believe that the current system for reporting CM is effective in addressing the problem	77	2.79	(1.030)	120	2.72	(0.688)
I would like to fulfill my professional responsibility by reporting suspected cases of child sexual abuse	78	4.53	(0.801)	120	4.52	(0.661)
I will consult with an administrator/supervisor before I report CM *	77	2.21	(0.951)	120	2.41	(1.141)
Reporting CM is necessary for the safety of children	78	4.64	(0.738)	120	4.60	(0.541)
CM reporting guidelines are necessary for physicians	77	4.52	(0.718)	119	4.33	(0.626)
It is important for physicians to be involved in reporting CM to prevent long-term consequences for children	77	4.45	(0.851)	120	4.43	(0.631)
Concerns about Reporting						
I would be apprehensive to report CM for fear of family retaliation *	78	3.67	(1.234)	120	2.61	(1.079)
I feel emotionally overwhelmed by the thought of reporting CM *	78	3.42	(1.254)	120	2.88	(1.006)
I would not report CM if I knew the child would be removed from the family/home *	78	3.94	(0.985)	116	2.23	(0.868)
I would consider not reporting CM because of the possibility of being sued *	78	4.17	(0.959)	120	2.49	(1.123)
There is a lot of sensitivity associated with reporting CM *	78	1.97	(0.980)	119	4.05	(1.431)
Physicians who report CM that is unsubstantiated can get in trouble *	77	3.55	(1.130)	120	3.18	(0.976)
It is a waste of time to report CM because no one will follow up on the report *	77	3.96	(1.081)	120	2.04	(0.911)
I would find it difficult to report CM because it is difficult to gather enough evidence *	78	3.37	(1.118)	120	3.09	(1.021)
A CM report can cause a parent to become more abusive toward the child *	78	2.78	(1.015)	120	3.58	(1.010)

Note. Item responses range from 1 to 5. Items without asterisks are scored as follows: strongly agree = 5, agree = 4, neutral = 3, disagree = 2, and strongly disagree = 1. Items with asterisks are scored as follows: strongly agree = 1, agree = 2, neutral = 3, disagree = 4, and strongly disagree = 5. Higher scores on the Reporting Responsibilities subscale items suggest better knowledge about and commitment to reporting CM. Higher scores on the Concerns about Reporting subscale indicate less apprehension about and fewer perceived obstacles to reporting CM.

**Table 2:** One-way ANOVA results.

	Reporting Responsibilities						Concerns about Reporting					
	*SS*	*df*	*MS*	*F*	*p*	*η^2^*	*SS*	*df*	*MS*	*F*	*p*	*η^2^*
Between groups	53.265	5	10.653	0.745	0.591	0.019	1646.798	5	329.360	11.564	<0.001	0.237
Within groups	2716.750	190	14.299				5297.572	186	28.482			

Note. ANOVA results using “Right now, how likely would you be to report CM if you suspected, but did not know for sure, that a child has been maltreated?” as the criterion. *SS* = sum of squares, *dF* = degrees of freedom, *MS* = mean square.

## Data Availability

Data sharing not applicable.
